# Rapid Detection of Chlorpheniramine Maleate in Human Blood and Urine Samples Based on NiCoP/PVP/PAN/CNFs Electrochemiluminescence Sensor

**DOI:** 10.3390/molecules30122603

**Published:** 2025-06-16

**Authors:** Yi Zhang, Jiayu Zhao, Jiaxing Chen, Tingfan Tang, Hao Cheng

**Affiliations:** 1 School of Biology, Food and Environment, Hefei University, Hefei 230601, China; yizhangkim@126.com (Y.Z.); 14755665491@163.com (J.C.); 2 Guangxi Key Laboratory of Green Processing of Sugar Resources, Guangxi Liuzhou Luosifen Research Center of Engineering Technology, College of Biological and Chemical Engineering, Guangxi University of Science and Technology, Liuzhou 545006, China; w15684190037@163.com (J.Z.); tangtingfan@163.com (T.T.); 3 Province and Ministry Co-Sponsored Collaborative Innovation Center of Sugarcane and Sugar Industry, Nanning 530004, China

**Keywords:** electrostatically spun film, porous carbon nanofiber, NiCoP, polyacrylonitrile, electrochemiluminescence

## Abstract

Chlorpheniramine maleate (CPM) is a first-generation antihistamine that is frequently used to treat allergic reactions. However, excessive consumption presents potential health risks. Therefore, it is crucial to develop a quick and precise technique for identifying CPM levels. In this study, nickel cobalt phosphide (NiCoP), a binary metal phosphide, was successfully incorporated into carbon nanofibers. This involved creating a pore structure by adding polyvinylpyrrolidone (PVP) as a pore-forming template to a polyacrylonitrile (PAN) substrate via electrostatic spinning. An innovative electrochemiluminescent sensor for CPM detection was constructed using NiCoP/PVP/PAN carbon nanofibers (NiCoP/PVP/PAN/CNFs). Under optimal conditions, the electrochemical behavior of CPM was studied using NiCoP/PVP/PAN/CNF-modified working electrodes. These findings demonstrate that the three-dimensional porous network architecture of NiCoP/PVP/PAN/CNFs enhances the conductive properties of the material. Consequently, an electrochemical optical sensor fabricated using this structure exhibited remarkable performance. The linear detection range of the sensor was 1 × 10^−8^–7 × 10^−5^ mol/L, and the detection limit was 7.8 × 10^−10^ mol/L. When human urine and serum samples were examined, the sensor was found to have a high recovery rate (94.35–103.36%), which is promising for practical applications.

## 1. Introduction

Chlorpheniramine maleate (CPM), also known as chlorpheniramine, is a first-generation antihistamine widely used to treat allergic reactions, hay fever, rashes, hives, and asthma [1]. In addition to being used as an anticholinergic in many over-the-counter combination drugs in parts of Asia, chlorpheniramine is also a common ingredient in many over-the-counter cold and cough medicines worldwide [2,3]. Abuse of chlorpheniramine is widespread owing to its legality, affordability, and easy availability. There have been many reports of chlorpheniramine overdose or toxicity, which usually begins with central nervous system depression, followed by euphoria, seizures, and convulsions [4]. In severe cases, it may lead to severe central nervous system depression, thereby posing a significant health risk. Recently, paracetamol has been reported to induce anaphylactic reactions in some populations. Treatment of anaphylactic reactions may lead to anaphylactic shock and clinical death [5]. Consequently, a method that can rapidly and sensitively detect CPM is urgently needed to detect drug overdoses and safeguard human health. Various methods for determining CPM levels have been reported in existing research, including tried-and-true techniques such as high-performance liquid chromatography (HPLC) [6] and reversed-phase HPLC (RP-HPLC) [7]. More advanced approaches, such as HPLC–tandem mass spectrometry (HPLC–MS/MS) [8] and HPLC ultraviolet detection (HPLC-UV) [9], have also been introduced to determine CPM levels.

Although these techniques are sensitive, they have several disadvantages as they require expensive instruments, preliminary treatment of the experimental samples, and extended operational times. Electrochemiluminescence (ECL) effectively circumvents these limitations by offering simplicity, cost-effectiveness, and rapid analysis [10], making it more suitable than other approaches for CPM testing. Loading suitable materials onto the surface of glassy carbon electrodes is a crucial step in the construction of novel electrochemiluminescent sensors because it enhances the sensitivity of CPM detection [11,12].

Carbon nanofibers (CNFs) have been shown to exhibit high temperature resistance, good chemical stability, and excellent electrical conductivity [13]. Electrostatic spinning is a simple and efficient method for the large-scale synthesis of carbon nanofibers and is widely reported in contemporary research. This method involves adjustment of the diameter for synthesizing nanofibers, yielding nanofibers with a range of morphologies and fine structural features [14]. In contemporary scientific research, preparing CNFs characterized by elevated specific surface areas and enhanced electrical conductivities via electrostatic spinning has emerged as a predominant research direction [15].

Electrospun fibers of polyacrylonitrile (PAN) [16], poly (vinylidene fluoride) [17], and phenolic resins [18] are frequently used in the fabrication of electrode materials. PAN is a notable precursor because of its affordability, excellent spinnability, high melting point, significant carbon yield, minimal polymerizable monomer content, and excellent processability. Therefore, it is widely used in the field of electrostatic spinning [19]. It has been demonstrated that PAN-based carbon fibers invariably possess low porosity and surface area owing to the polymer chain stacking during heat treatment [20]. Polyvinylpyrrolidone (PVP), a thermally unstable polymer, is frequently used as a template for the preparation of inorganic oxide nanofibers, which were eliminated after pyrolysis in ambient air. Previous studies indicated an increase in fiber porosity upon the introduction of PVP into polyacrylonitrile (PAN)-based materials [21].

In recent years, considerable attention has been paid to combining CNFs with battery-type materials, such as nickel- and cobalt-based oxides, hydroxides, sulfides, and phosphides, to form composites. These materials are notable for their high specific capacitances and energy densities, and the resulting composites exhibit enhanced electrochemical properties [22,23,24,25]. Transition-metal phosphides have emerged as a class of prospective electrodes displaying characteristics that include rich valence and properties reminiscent of metals [26]. Unitary phosphides such as NiP, Ni_2_P, and CoP have been the focus of extensive research [27,28,29]. The results demonstrate that incorporating a second metal element has a synergistic effect, providing more active sites, abundant redox reactions, and a higher specific capacitance than monophosphide. Notably, the favorable chemical stability and substantial theoretical value of the capacitance of nickel cobalt phosphide (NiCoP) manifest its exceptional electrochemical performance. However, pristine NiCoP, as an electrode modification material, suffers from relatively low conductivity, susceptibility to agglomeration, and poor stability [30]. Furthermore, existing methods for synthesizing phosphides entail intricate procedures that invariably result in the emission of toxic PH_3_ gases during the calcination process because of the incorporation of NaH_2_PO_2_ as a phosphorus source [31,32,33,34]. Methods with alternative phosphorus sources and improved reaction conditions have been proposed in existing studies to effectively reduce or avoid the production of PH_3_. For example, organic phosphorus sources have been utilized instead of conventional solid phosphate sources [35,36]. Consequently, it is imperative to identify a straightforward method to circumvent substantial NiCoP accumulation, thereby augmenting the properties of the composites and averting toxic emissions. Electrostatic spinning has emerged as a promising solution. There is a paucity of reports on the combination of NiCoP and CNFs as electrode modification materials. The integration of these two materials, leveraging the synergistic effect, offers numerous advantages, including enhanced conductivity, abundant charge transfer channels, expedited electrolytic ion diffusion pathways, a multitude of Faraday-reaction-accessible active sites, augmented surface area between the liquid electrolyte and active electrode material, and prevention of substantial agglomeration of NiCoP [37].

In this experiment, PVP was used as a pore-forming template in a PAN substrate via electrostatic spinning to generate a pore structure. NiCoP, a binary metal phosphide, was introduced into carbon nanofibers to form NiCoP/PVP/PAN/CNFs. These fibers possess several advantageous properties, including a large specific surface area and a well-developed pore structure. In addition, the nanofibers exhibit good electrical conductivity, which further enhances their functionality. Ternary carbon nanofibers were then used to assemble electrochemiluminescent sensors for CPM detection. These sensors offer several advantages over traditional methods, including enhanced sensitivity, selectivity, convenience, speed, and accuracy. Thus, the sensors excel in directly quantifying CPM in actual samples.

## 2. Results and Discussion

### 2.1. Characterization of Materials

**Figure 1:**
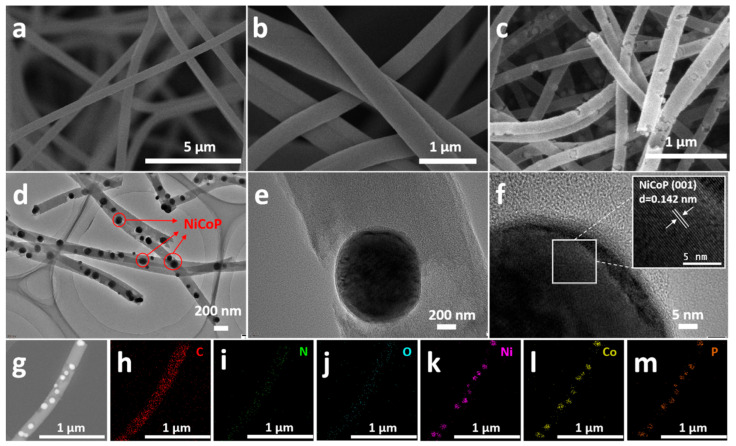
(**a**) SEM images of PAN/CNFs; (**b**) SEM images of PVP/PAN/CNFs; (**c**) SEM images of NiCoP/PVP/PAN/CNFs; (**d**–**g**) TEM images of NiCoP/PVP/PAN/CNFs; (**h**–**m**) EDS distribution of C, O, N, Ni, Co, P elements of NiCoP/PVP/PAN/CNFs.

The morphologies of PAN/CNFs, PVP/PAN/CNFs, and NiCoP/PVP/PAN/CNFs were characterized by SEM. Figure 1a shows that the PAN/CNFs had a continuous, uniform, and smooth two-dimensional fiber shape. At the same time, Figure 1b shows that some of the PVP/PAN/CNF fibers have a cross-linking appearance, mainly because PAN can form a stable structure during the carbonization process under the same pyrolysis conditions. In contrast, PVP exhibits severe atrophy due to melting during pyrolysis, which then leads to carbon nanofibers appearing as a cross-linked three-dimensional network structure, which not only increases the stability of the material but also increases the tension of the CNFs. As illustrated in Figure 1c, the synthesized NiCoP/PVP/PAN/CNFs material exhibited a pronounced porous surface architecture. This configuration markedly improves the surface area of the material and increases the number of active sites accessible to the reaction medium. High-resolution imagery distinctly revealed spherical NiCoP nanoparticles anchored onto carbon nanofibers (CNFs). This morphological evidence provides robust verification of the successful preparation of the NiCoP/PVP/PAN/CNFs composite.

TEM analysis revealed the characteristics of NiCoP/PVP/PAN/CNFs, as depicted in Figure 1d, which shows an obvious pore structure on the fiber surface, consistent with the SEM characterization results. Black particles were observed in the fibers, the color of which was very different from that of the carbon nanofibers. One of the spherical particles was magnified, and a lattice stripe of 0.142 nm, corresponding to the (001) crystal surface of NiCoP, was observed in Figure 1e,f. As shown in Figure 1g–m, NiCoP nanoparticles exhibited uniform dispersion within the CNFs, and the EDS characterization results showed that the NiCoP/PVP/PAN/CNFs were mainly composed of six elements, C, O, N, Ni, Co, and P, and the spherical particles in the CNFs were mainly composed of three elements, Ni, Co, and P, confirming the successful preparation of the NiCoP/PVP /PAN/CNFs.

**Figure 2:**
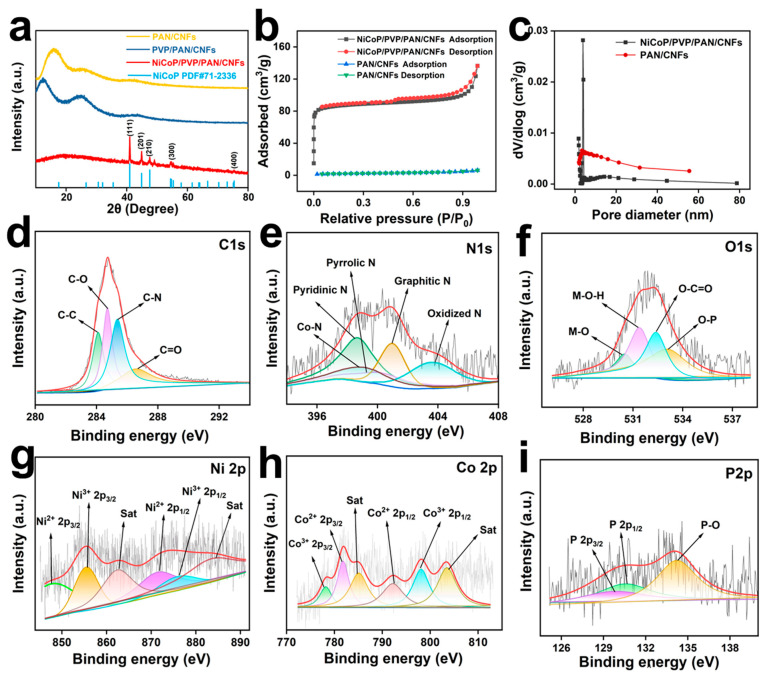
(**a**) XRD diffractograms of PAN/CNFs, PVP/PAN/CNFs, and NiCoP/PVP/PAN/CNFs; (**b**) N_2_ adsorption-desorption isotherms and (**c**) corresponding pore distributions of PAN/CNFs and PVP/PAN/CNFs; XPS characteristic peak spectra of (**d**) C1s, (**e**) N1s, (**f**) O1s, (**g**) Ni2p, (**h**) Co2p, and (**i**) P2p of NiCoP/PVP/PAN/CNFs.

XRD was used to analyze the crystal structures and phase compositions of the PAN/CNF, PVP/PAN/CNF, and NiCoP/PVP/PAN/CNF composites. Figure 2a illustrates a clear distinction in the diffraction patterns of NiCoP/PVP/PAN/CNFs, exhibiting unique peaks at 40.9°, 44.8°, 47.5°, and 54.4°. These angles correspond to the (111), (201), (210), and (300) crystallographic planes, respectively, and are aligned with NiCoP PDF number 71–2336 [38,39], indicating that NiCoP was successfully doped into the CNFs.

Figure 2b,c displays the porosity and surface characteristics of PAN/CNFs and NiCoP/PVP/PAN/CNFs, measured via N_2_ adsorption-desorption. Figure 2b reveals that the NiCoP/PVP/PAN/CNFs isotherms exhibit type IV behavior, with adsorption-desorption curves showing H4 hysteresis loop deviations [40]. The hysteresis loops in the Type IV isotherms are derived from capillary condensation within the mesopores, suggesting the presence of a mesoporous structure. Further, the H4 hysteresis loops are more pronounced at low relative pressures, where the separation of the adsorption and desorption curves is usually associated with narrow cleavage pore structures or mixed micro- and mesopore systems (e.g., activated carbons, layered porous materials). For NiCoP/PVP/PAN/CNFs, the appearance of H4-type hysteresis loops suggests that the pore structure may consist of narrow cleavage pores or micro-mesoporous synergisms, where the gas adsorbs through the multilayers to cover the pore walls at low pressures. As the pressure increases, the mesoporous region undergoes capillary coalescence, leading to characteristic hysteresis behavior [41,42]. This proves that NiCoP/PVP/PAN/CNFs are carbon nanofibers with a hierarchical pore structure in the presence of micropores and mesopores, which can be confirmed from the pore size distribution shown in Figure 2c. As depicted in Figure 2c, the pore size distribution in the NiCoP/PVP/PAN/CNF composite exhibited a concentration in the range of 0–20 nm, indicating a significant presence of both micropores and mesopores within the substance. These findings are consistent with the data derived from the N_2_ adsorption-desorption isotherm analysis, reinforcing the credibility of the methodology employed. NiCoP/PVP/PAN/CNFs exhibit a large surface area (393.44 m^2^/g) and a pore volume of 0.087 cm^3^/g. The increased specific surface area of the composite provided an increased number of accessible active sites, thereby contributing to the improved catalytic performance.

X-ray photoelectron spectroscopy (XPS) was employed to investigate the elemental composition and chemical states of the NiCoP/PVP/PAN/CNFs. As shown in Figure 2d, the C 1s spectrum reveals peaks corresponding to the C–C (284.4 eV), C–O (284.8 eV), C–N (285.6 eV), and C=O (287.4 eV) bonds [43,44], indicating the successful transformation of PAN and PVP into graphitic carbon during high-temperature carbonization. Figure 2e shows the N 1s spectrum, which includes pyridinic N (398.7 eV), Co–N (399.1 eV), pyrrolic N (399.6 eV), graphitic N (401.0 eV), and oxidized N (403.6 eV) species [45,46,47]. These nitrogen configurations suggest effective N doping within the carbon matrix, which promotes charge transfer and facilitates catalytic activity. As shown in Figure 2f, the O 1s spectrum presents signals from M–O (530.5 eV), M–OH (531.4 eV), O–C=O (532.4 eV), and O–P (533.1 eV) species (M=Ni, Co) [48,49], further confirming the occurrence of metal–oxygen and phosphorus–oxygen interactions. The Ni 2p spectrum (Figure 2g) reveals multiple oxidation states, with peaks assigned to Ni^2+^ 2p_1/2_ (870.5 eV), Ni^3+^ 2p_1/2_ (875.4 eV), and their satellite peaks (878.5 eV), as well as Ni^2+^ 2p_3/2_ (852.9 eV), Ni^3+^ 2p_3/2_ (857.3 eV), and associated satellites (860.5 eV). These species likely originate from surface oxidation upon exposure to air [30]. Figure 2h illustrates the Co 2p spectrum, featuring Co^2+^ 2p_1/2_ (792.2 eV), Co^3+^ 2p_1/2_ (798.1 eV), and corresponding satellites (803.5 eV), along with Co^2+^ 2p_3/2_ (781.8 eV), Co^3+^ 2p_3/2_ (778.2 eV), and satellites (785.1 eV), confirming the coexistence of mixed valence states of cobalt [50]. Finally, the P 2p spectrum displays peaks for P 2p_3/2_ (129.7 eV), P 2p_1/2_ (130.5 eV), and a higher binding energy peak at 134.2 eV corresponding to P–O bonds. The presence of P–O was ascribed to the surface phosphate species formed by the oxidation of phosphorus [51].

### 2.2. Electrochemistry and Electrochemiluminescence in the System

**Figure 3:**
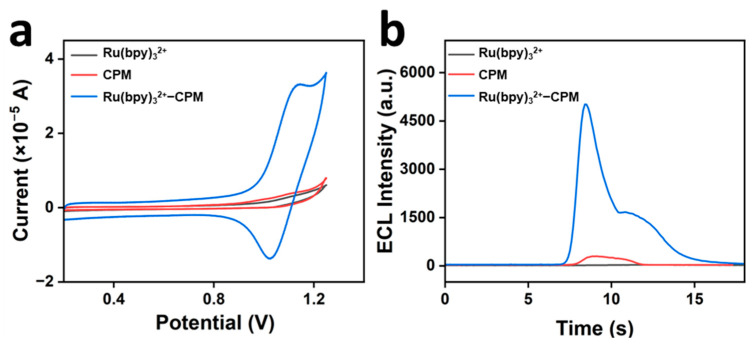
Electrochemical curves (**a**) and electrochemiluminescence curves (**b**) of CPM, Ru(bpy)_3_^2+^, and Ru(bpy)_3_^2+^−CPM systems.

Figure 3a,b illustrates the electrochemical and electrochemiluminescence activities of CPM, Ru(bpy)_3_^2+^, and Ru(bpy)_3_^2+^-CPM, respectively, on the modified electrodes. As shown in Figure 3a, the redox peak currents of the systems with only CPM and Ru(bpy)_3_^2+^ were inadequate. The oxidation and reduction peak currents of the system were substantially enhanced upon incorporation of Ru(bpy)_3_^2+^ into the CPM. This observation suggests that CPM underwent a redox reaction with Ru(bpy)_3_^2+^, thereby amplifying the electrochemical response within the system. As shown in Figure 3b, the electrochemical luminescence signal of the CPM-only system was remarkably weak, and the Ru(bpy)_3_^2+^−only system exhibited an almost negligible electrochemical luminescence signal. However, the luminescence intensity was substantially enhanced upon the incorporation of CPM into the Ru(bpy)_3_^2+^ system. This observation indicated that CPM exerted a pronounced sensitizing effect on the luminescence of the Ru(bpy)_3_^2+^ system. The reaction mechanism is shown in Equations (1)–(5)

(1) $$\mathrm{Ru}{\left(\mathrm{bpy}\right)}_{3}^{2+}-e^{-}\to \mathrm{Ru}{\left(\mathrm{bpy}\right)}_{3}^{3+}$$

(2) $$\mathrm{CPM}-e^{-}\to {\mathrm{CPM}*}^{+}$$

(3) $${\mathrm{CPM}*}^{+}\to {\mathrm{CPM}}^{*}+H^{+}$$

(4) $$\mathrm{Ru}{\left(\mathrm{bpy}\right)}_{3}^{3+}+\mathrm{CPM}*\to \left[\mathrm{Ru}{\left(\mathrm{bpy}\right)}_{3}^{2+}\right]*+{\mathrm{CPM}}^{+}$$

(5) $$\left[\mathrm{Ru}{\left(\mathrm{bpy}\right)}_{3}^{2+}\right]*\to \mathrm{Ru}{\left(\mathrm{bpy}\right)}_{3}^{2+}+h\nu$$

**Figure 4:**
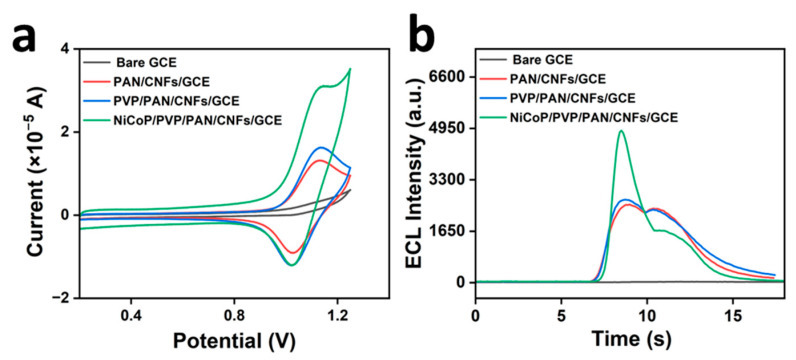
CV (**a**) and ECLs (**b**) of the Ru(bpy)_3_^2+^−CPM system on different modified electrodes.

Figure 4a,b shows the CV and ECL curves of the Ru(bpy)_3_^2+^-CPM system on the bare electrode, PAN/CNFs/glassy carbon electrodes (GCEs), PVP/PAN/CNFs/GCE, and NiCoP/PVP/PAN/CNFs/GCE, respectively. As shown in Figure 4a, the Ru(bpy)_3_^2+^−CPM system’s redox peak signals were noticeably stronger on the NiCoP/PVP/PAN/CNFs/GCE-modified electrodes than on the other electrodes. This enhancement is attributed to the NiCoP in the NiCoP/PVP/PAN/CNFs/GCE, which is a good conductor of electricity and increases the intensity of the CV signals. As shown in Figure 4b, the Ru(bpy)_3_^2+^−CPM system’s electrochemical luminance increased when using the NiCoP/PVP/PAN/CNF/GCE-coated electrode. This implies that the modified electrode supported the Ru(bpy)_3_^2+^−CPM system. This enhancement is attributed to the material’s highly porous architecture and substantial specific surface area, which afford an abundant supply of active sites for facilitating reaction kinetics, thereby enhancing signal transduction efficiency.

### 2.3. Experimental Parameter Refinement

#### 2.3.1. Effect of Modification Amount of NiCoP/PVP/PAN/CNFs

**Figure 5:**
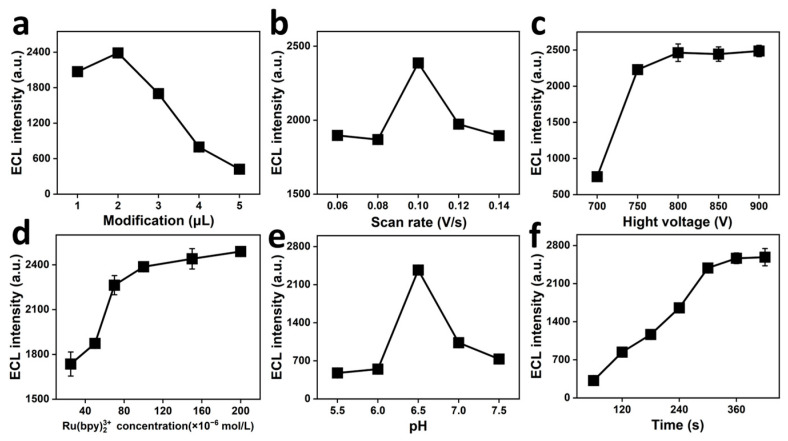
Effect of NiCoP/PVP/PAN/CNFs modification amount (**a**), scanning rate (**b**), photomultiplier high pressure (**c**), Ru(bpy)_3_^2+^ concentration (**d**), pH (**e**), and enrichment time (**f**) on the ECL intensity.

This study examined the effect of altering the NiCoP/PVP/PAN/CNF loading on electrochemiluminescence. As demonstrated in Figure 5a, with the increasing amount of NiCoP/PVP/PAN/CNFs modification, the ECL signal exhibited an initial increase and subsequent decrease, reaching a maximum intensity at the modification amount of 2 μL. This was preceded by an increase in the number of NiCoP/PVP/PAN/CNFs, causing an increase in the number of active sites for the reaction, which accelerated the redox reaction. The signal started to dip once more than 2 μL had built up. This is mainly because the modified film on the electrode became too thick with continued addition. As the film thickened, it obstructed the electron transfer process on the surface of the electrode. This, in turn, obstructed the redox reaction, causing the ECL signal to diminish.

#### 2.3.2. Effect of Scan Rate

This study focuses on how the sweep rate affects the ECL intensity. In Figure 5b, you can see that the ECL intensity peaks at a sweep rate of 0.10 V/s, specifically when discussing sweep rates ranging from 0.06 to 0.14 V/s. This phenomenon is primarily attributed to the progressively active production and annihilation rates of the excited state [Ru(bpy)_3_^2+^]* within the range of 0.06–0.10 V/s. As the scanning rate increases, the ECL signal generated at the electrode surface equilibrates with the specific surface area of the electrode at 0.10 V/s, which leads to the maximum ECL signal. As the scan rate increases, the reaction equilibrium at the electrode surface is disrupted, resulting in a decrease in the ECL signal.

#### 2.3.3. Effect of Photomultiplying High Voltage

The ECL intensity of the photomultiplier voltage at 650–900 V was examined. As shown in Figure 5c, an increase in the photomultiplier voltage from 700 to 800 V resulted in a gradual increase in the peak current, which reached its maximum at 800 V. It is important to note that when the photomultiplier voltage exceeds 800 V, there is no substantial improvement in the ECL signal when the voltage is further increased. This was because prolonging the service life of the photomultiplier tube and maintaining the stability of the detected signals were more effectively achieved by selecting an optimal photomultiplier voltage of 800 V.

#### 2.3.4. Impact of Ru(bpy)_3_^2+^ Concentration

The present study investigated the effect of the Ru(bpy)_3_^2+^ concentration on the ECL signal. As demonstrated in Figure 5d, Ru(bpy)_3_^2+^ concentrations ranging from 2.5 × 10^−5^ to 2.0 × 10^−4^ mol/L enhanced the ECL intensity and subsequently attained stability. ECL peaked at 1.0 × 10^−4^ mol/L, then stabilized despite rising Ru(bpy)_3_^2+^ concentrations. This could be due to the balance that is struck in the Ru(bpy)_3_^2+^ concentration in its high-valent state on the electrode surface, beyond which adding more will not boost the ECL signal strength. Given the high cost of Ru(bpy)_3_^2+^, we determined that 1.0 × 10^−4^ mol/L of Ru(bpy)_3_^2+^ was the optimal experimental concentration.

#### 2.3.5. Effect of pH Value

This study investigated the effect of pH on the ECL strength. As depicted in Figure 5e, the ECL brightness in the PBS buffer system saw a surge within the pH spectrum of 5.5 to 7.5, only to taper off after that, peaking at pH 6.5. A reduction in pH was observed to influence the activity of Ru(bpy)_3_^2+^ in the reaction, resulting in the enhancement of the ECL signal during periods of acidity decline. It has been demonstrated that an increase in alkalinity can result in a reaction between the OH- group and Ru(bpy)_3_^2+^. This reaction decreases the Ru(bpy)_3_^2+^ concentration in the electrolyte, thereby reducing its capacity to react with CPM. Consequently, this decrease in Ru(bpy)_3_^2+^ concentration results in a decline in the ECL signal.

#### 2.3.6. Effect of Enrichment Time

This study investigated the effect of enrichment time on the ECL intensity. As shown in Figure 5f, increasing the enrichment time from 60 to 300 s enhanced the ECL intensity. These findings suggest a notable increase in LIN accumulation on the electrode face, consequently boosting the redox interactions between CPM and Ru(bpy)_3_^2+^. The enhancement in the electrochemiluminescence signal was significant [36]. After 300 s, the negligible increase in the ECL intensity with time suggests that the CPM loaded on the electrode surface attained saturation [37]. Consequently, the enrichment time was set at 300 s in this study.

### 2.4. Methodological Evaluations

#### 2.4.1. Establishment of the Linear Equation

**Figure 6:**
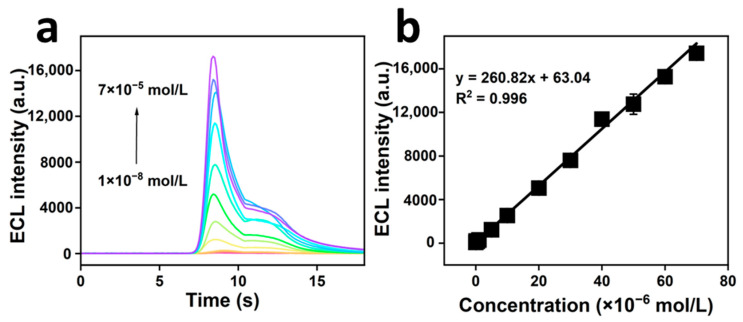
ECL curves (**a**) and standard curves (**b**) at CPM concentrations from 1.0 × 10^–8^ to 7.0 × 10^–5^ mol/L under optimal experimental conditions (n = 3).

The change rule of the ECL signal after altering the CPM concentration under optimal experimental conditions is illustrated in Figure 6a,b. As illustrated in Figure 6a, the ECL signal intensity exhibited consistent enhancement with increasing CPM concentration. Linear regression analysis was performed using the ECL signal intensity against the CPM concentration. As illustrated in Figure 6b, the ECL signal intensity (y) exhibited a substantial linear relationship with the CPM concentration (x) when the CPM concentration ranged from 1.0 × 10^−8^ to 7.0 × 10^−5^ mol/L. The linear equation obtained was y = 260.82x + 63.04, with a linear correlation coefficient (R²) of 0.996 and limit of detection (LOD) of 7.8 × 10^−10^ mol/L. These results highlight the robust relationship between the ECL signal intensity (y) and CPM concentration (x).

As illustrated in Table 1, the CPM assay developed in this study exhibited a low detection limit and a broad linear range compared to other assays.

#### 2.4.2. Selectivity, Repeatability, and Reproducibility of NiCoP/PVP/PAN/CNFs Modified Electrodes

**Figure 7:**
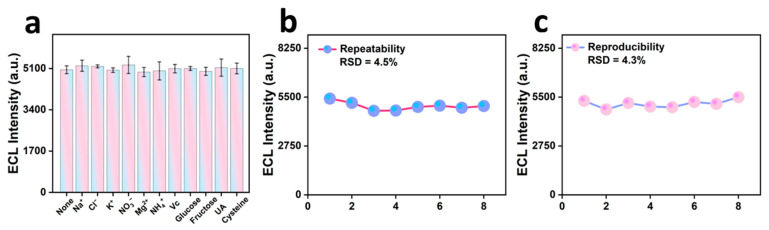
Selectivity (**a**), repeatability (**b**), and reproducibility (**c**) of NiCoP/PVP/PAN/CNFs (*n* = 3).

The anti-interference ability of the NiCoP/PVP/PAN/CNFs and the specific detection of CPM were investigated. We first measured the ECL signals of the CPM system alone, followed by experiments in which different concentrations of interfering ions were separately added to the system. In a 100 μL solution of 20 μM CPM, Na^+^, Cl^−^, K^+^, NO_3_^−^, and Mg^2+^ were added at concentrations 500 times that of CPM; 300 times that of NH_4_^+^; 30 times that of ascorbic acid (Vc) and uric acid (UA); 20 times that of glucose and fructose; and 10 times that of cysteine (Cys). As shown in Figure 7a, the electrochemiluminescence intensity of NiCoP/PVP/PAN/CNFs in the blank group to that in the control group was less than 5%. This indicates that the coexisting compounds had no significant effect on the detection of CPM.

The repeatability and reproducibility of the NiCoP/PVP/PAN/CNFs electrochemical method for photosensors were evaluated. We conducted the experiments with reference to the literature [57]. Under the same experimental conditions, the same instrument, operator, and working electrode loaded with NiCoP/PVP/PAN/CNFs were used for the eight CPM assays, and the relative standard deviation (RSD) of the eight measurements was approximately 4.5%, which indicated that the good reproducibility of the modified electrode. Similarly, we performed eight separate assays to measure the ECL intensity of CPM at different times using different operators and eight different electrodes loaded with NiCoP/PVP/PAN/CNFs. In these experiments, the RSD of ECL intensity increased by approximately 4.3%. This suggests that the electrodes that we fabricated were consistent in their performance.

### 2.5. Examination of Authentic Samples

Table 2 presents the analytical results obtained from the authentic samples. The recovery rate for CPM decreased from 94.35% to 103.36% with a relative standard deviation of less than 6%. This suggests that the modified electrode is promising for detecting CPM in real-world samples. Comparative experiments were performed using high-performance liquid chromatography and electrochemiluminescence methods. The detection results obtained by the ECL method showed good consistency with those of HPLC. This finding further validates the applicability of NiCoP/PVP/PAN/CNFs electrochemiluminescence sensors for the analysis of real-world samples.

## 3. Experimental Section

### 3.1. Experimental Reagents

Supplementary Material Text S1 presents the experimental reagents, chemicals, and analytical instruments used in the experiment.

### 3.2. Preparation of NiCoP/PVP/PAN/CNFs Material

**Scheme 1:**
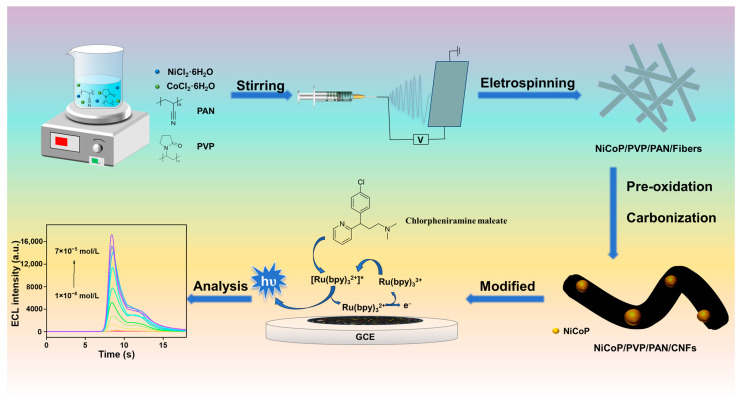
Synthesis of NiCoP/PVP/PAN/CNFs, assembly of ECL sensors, and applications.

Scheme 1 shows a schematic diagram of the synthesis of NiCoP/PVP/PAN/CNFs nanofibers, which is a feasible method for avoiding PH_3_ emission during preparation. First, PAN (0.5 g) and PVP (0.5 g) were dissolved in dimethylformamide (DMF, 10 mL) at room temperature. Then, 0.238 g NiCl_2_·6H_2_O, 0.142 g CoCl_2_·6H_2_O, and 0.238 g PPA were added to the above solution and stirred overnight to prepare the precursor in the NiCoP/PVP/PAN manner. The obtained precursor solution was then transferred to a syringe, an aluminum foil paper was wound on a roller, and the distance from the needle to the roller was fixed at 13.2 cm. The solution was then electrostatically spun under high voltage (11.3 kV at a feed rate of 1.5 cm/h). The obtained NiCoP/PVP/PAN nanofiber mats are referred to as NiCoP/PVP/PAN fibers.

The NiCoP/PVP/PAN composite fibers were subjected to a multistep thermal treatment. First, they were dried in a vacuum oven at 60 °C for 12 h to remove moisture. The material was then heated up to 250 °C in a muffle furnace at a ramping rate of 2 °C/min and stabilized at this temperature for two hours to enhance its structural integrity. The final step was carried out in a tube furnace under nitrogen at a ramp rate of 5 °C/min up to 600 °C, allowing the material to phosphatize under these conditions for two hours. This carefully controlled thermal process yielded the desired NiCoP/PVP/PAN carbon nanofiber composite (NiCoP/PVP/PAN/CNFs).

### 3.3. Preparation of NiCoP/PVP/PAN/CNFs/GCE

The glassy carbon electrode (GCE) was polished with 0.5 μm alumina powder on the chamois leather, and the polished GCE was ultrasonically cleaned with water and ethanol in an ultrasonic machine and blow-dried with N_2_ as a reserve. The prepared NiCoP/PVP/PAN/CNFs were ground in an agate mortar, and 1 mg of the powder was weighed, added to 1 mL of the dispersion solution, and ultrasonicated for 30 min to obtain the NiCoP/PVP/PAN/CNFs suspension. The dispersion solution was composed of isopropanol, ultrapure water, and Nafion solution and was added in volume ratio, V_isopropanol_:V_ultrapure water_:V_Nafion_ = 16:4:1. The dispersion was pipetted with a liquid pipette gun to modify the GCE, and the modified GCE was exposed to air to dry naturally. A NiCoP/PVP/PAN/CNF-modified electrode was prepared and designated NiCoP/PVP/PAN/CNFs/GCE.

For comparison, the same concentrations of PAN/CNF and PVP/PAN/CNF suspensions were configured, and the GCE was treated under the same conditions to obtain PAN/CNF/GCE and PVP/PAN/CNF/GCE, respectively.

### 3.4. Electrochemical and ECL Analysis

The detailed electrochemical parameters, electrochemiluminescence data, electrolyte preparation methods, and electrode selection criteria used in the experiments are provided in Supplementary Material Text S2.

### 3.5. Handling of Real Samples

Blood serum samples were provided by Chengdu Shilian Kangjian Biotechnology Co., Ltd. (Chengdu, China), and urine samples were provided by volunteers and kept promptly refrigerated. The blood serum samples underwent centrifugation at 4000 revolutions per minute for half an hour while being maintained at 4 °C. Following this, the liquid portion was carefully passed through a 0.22 micron PVDF membrane filter. For analysis, a 1 mL aliquot of the processed blood serum samples was mixed with phosphate-buffered saline (pH 6.5) to create a 50 mL solution. Unlike the blood samples, the urine specimens were used directly without preliminary preparation.

High-performance liquid chromatography (HPLC) was used to validate the results of the NiCoP/PVP/PAN/CNFs electrochemical method for the detection of CPM by the photosensor. A high-performance liquid chromatograph equipped with Shim-pack GIST C18 5 μm as the detection column was selected for the detection of CPM, and the detection wavelength was set at 225 nm to achieve specific monitoring of the target drugs. The serum samples were filtered through a 0.22 μm filter membrane, and HPLC detected the filtrate. A butter of 0.05 M hydrogen phosphate dibasic salt at pH = 4 was used, and methanol–phosphate = 30:70 was selected as the mobile phase for the detection of CPM, and the column temperature was set at 30 °C, the injection rate was set at 1 mL/min, and the injection volume was 20 μL. The detection of CPM was carried out at a flow rate of 5 × 10^−7^ mol/L, 1 × 10^−6^ mol/L, 1 × 10^−5^ mol/L, 2 × 10^−5^ mol/L, 2.5 × 10^−5^ mol/L, and 3.5 × 10^−5^ mol/L to establish a standard curve for HPLC analysis of CPM.

## 4. Conclusions

In this study, a composite material, NiCoP/PVP/PAN/CNFs, was synthesized using electrostatic spinning. These composites were used as GCE. The unique chemiluminescence and electroluminescence properties exhibited by NiCoP/PVP/PAN/CNFs on CPM were thoroughly examined under optimal conditions. NiCoP/PVP/PAN/CNFs exhibited advantageous electrochemical properties, enhancing the electrical conductivity of the material and augmenting the ECL signal intensity. Moreover, the intricate network of the NiCoP/PVP/PAN/CNF composite offered a rich tapestry of active sites, which in turn boosted the amplitude of the electrochemical signal. Its superior surface characteristics amplified the redox activity within the reaction ensemble, thereby increasing the sensitivity of the chemiluminescent detection signal. A CPM electrochemiluminescence sensor that is rapid, straightforward in operation, and highly sensitive was successfully constructed based on the favorable properties of NiCoP/PVP/PAN/CNFs. The sensor demonstrated low detection thresholds, consistent and reproducible results, stability, and high selectivity, making it suitable for accurately identifying actual CPM samples.

## Figures and Tables

**Table 1:** Comparison of various methodologies employed for the detection of CPM.

Method	Linear Range/(mol/L)	LOD/(mol/L)	Reference
UHPLC-MS/MS	2.5 × 10^−5^–6.3 × 10^−4^	1.2 × 10^−5^	[52]
RP-HPLC	1.2 × 10^−5^–7.6 × 10^−5^	1.6 × 10^−9^	[53]
DCBI-MS	1.2 × 10^−5^–1.2 × 10^−4^	5.1 × 10^−7^	[54]
HPLC-UV	5.1 × 10^−8^–1.4 × 10^−6^	1.0 × 10^−9^	[54]
SP	6.4 × 10^−8^–3.2 × 10^−7^	3.8 × 10^−8^	[55]
DPV	1.0 × 10^−5^–6.0 × 10^−5^	6.2 × 10^−8^	[56]
ECL	1.0 × 10^−8^–7.0 × 10^−5^	7.8 × 10^−10^	This work

**Table 2:** Detection and recovery of CPM in real samples.

Detection Methods	Actual Samples	Add/(μmol/L)	Found ± SD/(μmol/L)	Recovery Rate/(%)	RSD/(%, *n* = 3)
ECL	Blood serum sample	0	-	-	-
		1	1.03 ± 0.05	103.36	5.48
		11	10.46 ± 0.49	95.09	4.70
		31	30.22 ± 0.08	97.51	0.29
	Urine sample	0	-	-	-
		1	1.01 ± 0.04	101.81	4.30
		11	10.37 ± 0.13	94.35	1.27
		31	29.85 ± 0.25	96.31	0.86
HPLC	Blood serum sample	0	-	-	-
		11	10.50 ± 0.07	95.45	1.23
		31	31.17 ± 0.32	100.54	3.22

## Data Availability

The data presented in this study are available on request from the corresponding author.

## Supplementary Materials

The following supporting information can be downloaded at: https://www.mdpi.com/article/10.3390/molecules30122603/s1, TEXT S1: Experimental Reagents; TEXT S2: Electrochemical and ECL Analysis; Figure S1: XPS gross spectra of NiCoP/PVP/PAN/CNFs; Figure S2: ECS diagram for NiCoP/PVP/PAN/CNFs; Figure S3: EL diagram of NiCoP/PVP/PAN/CNFs; Figure S4: NiCoP/PVP/PAN/CNFs modification solution (a) uniformly dispersed, (b) not uniformly dispersed.
