# Supplementary material for: Rapid Detection of Chlorpheniramine Maleate in Human Blood and Urine Samples Based on NiCoP/PVP/PAN/CNFs Electrochemiluminescence Sensor
Source: Molecules. 2025 Jun 16;30(12):2603. doi: 10.3390/molecules30122603 (PMC12196095; doi:10.3390/molecules30122603)
Supplement: Supplementary file 1 [file molecules-30-02603-s001.zip › molecules-3649459-supplementary.pdf]

Supporting Information

Rapid detection of chlorpheniramine maleate in human blood and urine samples based on NiCoP/PVP/PAN/CNFs electrochemiluminescence sensor

Yi Zhang[1], Jiayu Zhao[2], Jiaxing Chen[1], Tingfan Tang[2,3], Hao Cheng[*2,3]

1 School of Biology, Food and Environment, Hefei University, Hefei 230601, China; zhangtie@hfuu.edu.cn

2 Guangxi Key Laboratory of Green Processing of Sugar Resources, Guangxi Liuzhou Luosifen Research Center of Engineering Technology, College of Biological and Chemical Engineering, Guangxi University of Science and Technology, Liuzhou 545006, Guangxi, China

3 Province and Ministry Co-sponsored Collaborative Innovation Center of Sugarcane and Sugar Industry, Nanning 530004, Guangxi, China

* Corresponding authors: Guangxi Key Laboratory of Green Processing of Sugar Resources, College of Biological and Chemical Engineering, Guangxi University of Science and Technology, Liuzhou, 545006, Guangxi province, PR China. chenghao@gxust.edu.cn

TEXT S1

Polyacrylonitrile (PAN, Mw=150,000 g/mol), Polyvinylpyrrolidone (PVP, Mw=1,300,000 g/mol), Nickel chloride ($NiCl_2 \cdot 6H_2O$, AR), and Cobalt chloride ($CoCl_2 \cdot 6H_2O$, AR). The chemicals phenylphosphinic acid (PPA, 98%), disodium phosphate (Na_2HPO_4, AR), and sodium dihydrogen phosphate (NaH_2PO_4, AR) were procured from Shanghai McLean Biochemical Science and Technology Company. At the same time, the 5 wt% Nafion solution was obtained from Shanghai Bofamc Chemical Science and Technology Co. None of the reagents employed in the experimental setting underwent further purification. The water utilized in the experimental process was ultrapure.

The following advanced analytical instruments were employed in this study: SU8220 ultra-high-resolution cold field emission scanning electron microscope (SEM) manufactured by Nippon Electron Co., Ltd., and Thermoescalab 250Xi X-ray photoelectron spectrometer (XPS) from Thermo Fisher Scientific (USA) for surface chemical analysis. D8 ADVANCE Bruker X-ray diffractometer (XRD, Bruker, Germany) for crystallographic characterization. ASAP 2460 surface area and porosity analyser (BET method) supplied by Mack (USA) to determine specific surface area. MPI-E electrochemiluminescence analyser produced by Xi'an Ruimai Analytical Instruments Co., Ltd. Electrochemical measurements were conducted using a three-electrode system, consisting of: Ag/AgCl reference electrode； Pt wire counter electrode, ； Modified glassy carbon working electrode (GCE, 3 mm diameter) sourced from Tianjin Lanco Chemical & Electronic High-Tech Co., Ltd.

TEXT S2

The experimental setup employed a three-electrode configuration, with either a bare or modified glassy carbon electrode (GCE) as the working electrode. A platinum (Pt) electrode functioned as the counter electrode, while an Ag/AgCl electrode provided the reference potential. Electrochemical evaluations such as cyclic voltammetry (CV) and electrochemiluminescence (ECL) analyses were performed within a 0.1 M phosphate-buffered saline (PBS) mixture, enriched with 100 μL of $Ru(bpy)_3^{2+}$ to act as a glowy probe.

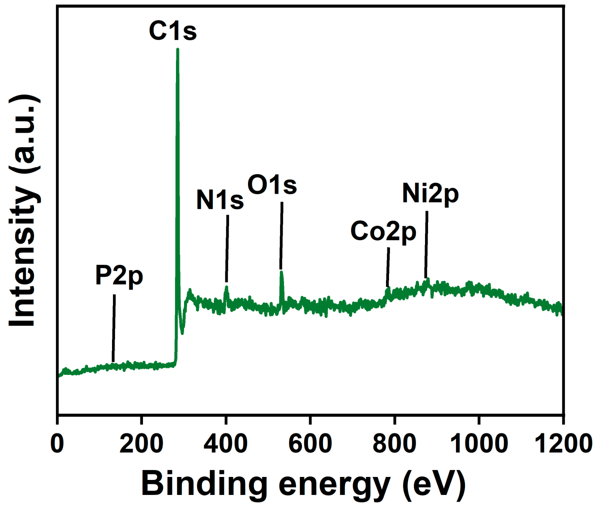

Figure S1. XPS gross spectra of NiCoP/PVP/PAN/CNFs

The elemental composition and chemical states of NiCoP/PVP/PAN/CNFs were analysed using XPS technique. As shown in Fig. S1, at 284.77 eV, 400.35 eV, 532 eV, 782.04 eV, 853.94 eV, and 134.51 eV, corresponding to C1s, N1s, O1s, Co2p, Ni2p, and P2p, respectively. the results indicate that the material is mainly composed of six elements, namely, C, N, O, Co, Ni, and P, with contents of 83.12%, 6.2%, 7.33%, 1.08%, 1%, 1.27%. This proves the successful synthesis of the material.

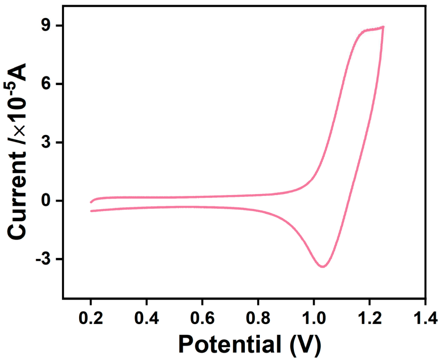

Figure S2. ECS diagram for NiCoP/PVP/PAN/CNFs

Figure S2 is the ECS plot of NiCoP/PVP/PAN/CNFs. From this figure, it is shown that there is an oxidation peak at +1.18 V and a reduction peak at +1.05 V. This phenomenon confirms the redox reaction of $Ru(bpy)_3^{2+}$ with CPM.

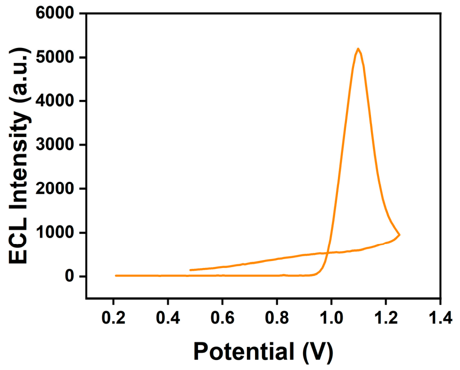

Figure S3. EL diagram of NiCoP/PVP/PAN/CNFs

Fig. S3 shows the EL diagram of NiCoP/PVP/PAN/CNFs, from which it can be seen that at a voltage of +0.95 V, the material starts to show electrochemical law light signals, and the luminescence intensity increases abruptly when the potential is scanned to + 1.05 V, forming a sharp luminescence peak, which corresponds to the generation of the luminescent excited state in the process of electrochemical oxidation, which corresponds to the oxidation peak in the ECS diagram. The luminescence originates from the excitation of $Ru(bpy)_3^{2+}$ on the electrode surface by the oxidation-reduction cycle. The overall results confirm that the ECL system possesses the characteristics of high sensitivity, fast response and stable luminescence.

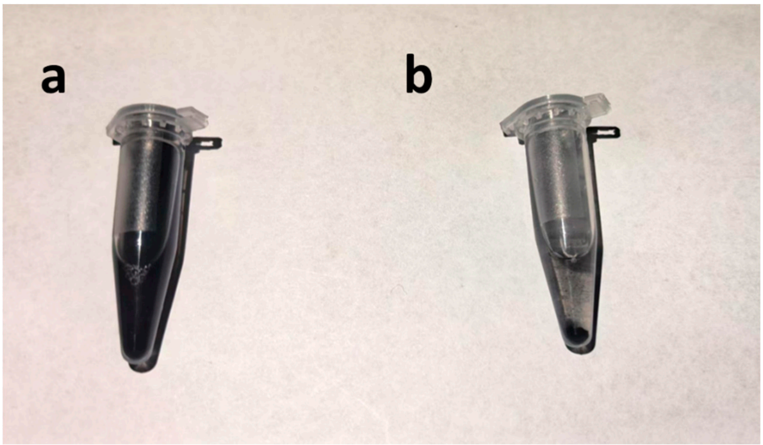

Figure S4. NiCoP/PVP/PAN/CNFs modification solution (a) uniformly dispersed, (b) not uniformly dispersed

Fig. S4 is a comparison of NiCoP/PVP/PAN/CNFs dispersions, where Fig. S4a shows that after 30 minutes of sonication of NiCoP/PVP/PAN/CNFs modifiers, a uniform dispersion can be observed. Figure S4b shows the NiCoP/PVP/PAN/CNFs modifier without sonication, where it can be observed that the NiCoP/PVP/PAN/CNFs are mainly concentrated at the bottom of the centrifuge tube and are not dispersed.
